# Coronavirus Disease among Workers in Food Processing, Food Manufacturing, and Agriculture Workplaces

**DOI:** 10.3201/eid2701.203821

**Published:** 2021-01

**Authors:** Michelle A. Waltenburg, Charles E. Rose, Tristan Victoroff, Marilee Butterfield, Jennifer A. Dillaha, Amy Heinzerling, Meagan Chuey, Maria Fierro, Rachel H. Jervis, Kristen M. Fedak, Andrea Leapley, Julie A. Gabel, Amanda Feldpausch, Eileen M. Dunne, Connie Austin, Caitlin S. Pedati, Farah S. Ahmed, Sheri Tubach, Charles Rhea, Julius Tonzel, Anna Krueger, David A. Crum, Johanna Vostok, Michael J. Moore, Hannah Kempher, Joni Scheftel, George Turabelidze, Derry Stover, Matthew Donahue, Deepam Thomas, Karen Edge, Bernadette Gutierrez, Erica Berl, Meagan McLafferty, Kelly E. Kline, Nichole Martz, James C. Rajotte, Ernest Julian, Abdoulaye Diedhiou, Rachel Radcliffe, Joshua L. Clayton, Dustin Ortbahn, Jason Cummins, Bree Barbeau, Stacy Carpenter, Julia C. Pringle, Julia Murphy, Brandy Darby, Nicholas R. Graff, Tia K.H. Dostal, Ian W. Pray, Courtney Tillman, Dale A. Rose, Margaret A. Honein, CDC COVID-19 Emergency Response Team

**Affiliations:** Centers for Disease Control and Prevention, Atlanta, Georgia, USA (M.A. Waltenburg, C.E. Rose, T. Victoroff, M. Chuey, E.M. Dunne, M. Donahue, J.C. Pringle, I.W. Pray, D.A. Rose, M.A. Honein);; Arizona Department of Health Services, Phoenix, Arizona, USA (M. Butterfield);; Arkansas Department of Health, Little Rock, Arkansas, USA (J.A. Dillaha);; California Department of Public Health, Richmond, California, USA (A. Heinzerling);; County of San Diego Health and Human Services Agency, San Diego, California, USA (M. Chuey);; Imperial County Public Health Department, El Centro, California, USA (M. Fierro);; Colorado Department of Public Health and Environment, Denver, Colorado, USA (R.H. Jervis, K.M. Fedak);; Florida Department of Health, Tallahassee, Florida, USA (A. Leapley);; Georgia Department of Public Health, Atlanta (J.A. Gabel, A. Feldpausch);; Idaho Department of Health and Welfare, Boise, Idaho, USA (E.M. Dunne);; Illinois Department of Public Health, Springfield, Illinois, USA (C. Austin);; Iowa Department of Public Health, Des Moines, Iowa, USA (C.S. Pedati);; Kansas Department of Health and Environment, Topeka, Kansas, USA (F.S. Ahmed, S. Tubach);; Kentucky Department for Public Health, Frankfort, Kentucky, USA (C. Rhea);; Louisiana Department of Health, New Orleans, Louisiana, USA (J. Tonzel);; Maine Center for Disease Control and Prevention, Augusta, Maine, USA (A. Krueger);; Maryland Department of Health, Baltimore, Maryland, USA (D.A. Crum);; Massachusetts Department of Public Health, Boston, Massachusetts, USA (J. Vostok, M.J. Moore);; Minnesota Department of Health, St. Paul, Minnesota, USA (H. Kempher, J. Scheftel);; Missouri Department of Health and Senior Services, Jefferson City, Missouri, USA (G. Turabelidze);; Nebraska Department of Health and Human Services, Lincoln, Nebraska, USA (D. Stover, M. Donahue);; New Jersey Department of Health, Trenton, New Jersey, USA (D. Thomas);; New Mexico Department of Health, Santa Fe, New Mexico, USA (K. Edge, B. Gutierrez);; North Carolina Department of Health and Human Services, Raleigh, North Carolina, USA (E. Berl);; Oregon Health Authority, Portland, Oregon, USA (M. McLafferty);; Pennsylvania Department of Health, Harrisburg, Pennsylvania, USA (K.E. Kline);; Pennsylvania Department of Agriculture, Harrisburg (N. Martz);; Rhode Island Department of Health, Providence, Rhode Island, USA (J.C. Rajotte, E. Julian);; South Carolina Department of Health and Environmental Control, Columbia, South Carolina, USA (A. Diedhiou, R. Radcliffe);; South Dakota Department of Health, Pierre, South Dakota, USA (J.L. Clayton, D. Ortbahn);; Tennessee Department of Health, Nashville, Tennessee, USA (J. Cummins);; Utah Department of Health, Salt Lake City, Utah, USA (B. Barbeau);; Vermont Department of Public Health, Burlington, Vermont, USA (S. Carpenter, J.C. Pringle);; Virginia Department of Health, Richmond, Virginia, USA (J. Murphy, B. Darby);; Washington State Department of Health, Shoreline, Washington, USA (N.R. Graff, T.K.H. Dostal);; Wisconsin Department of Health Services, Madison, Wisconsin, USA (I.W. Pray);; Wyoming Department of Health, Cheyenne, Wyoming, USA (C. Tillman)

**Keywords:** occupational health, worker safety, respiratory infections, severe acute respiratory syndrome coronavirus 2, SARS-CoV-2, SARS, COVID-19, coronavirus disease, zoonoses, viruses, coronavirus

## Abstract

We describe coronavirus disease (COVID-19) among US food manufacturing and agriculture workers and provide updated information on meat and poultry processing workers. Among 742 food and agriculture workplaces in 30 states, 8,978 workers had confirmed COVID-19; 55 workers died. Racial and ethnic minority workers could be disproportionately affected by COVID-19.

High-density workplaces can cause high risk for transmission of severe acute respiratory syndrome coronavirus 2 (SARS-CoV-2), the virus that causes coronavirus disease (COVID-19) (*1*–*3*). US food processing, food manufacturing, and agriculture workplaces employ >3.6 million persons (*4*). Several factors contribute to workplace and community transmission, including prolonged close contact with coworkers, congregate housing, shared transportation, and frequent community contact among workers (*1*,*2*). Prior reports have characterized COVID-19 among meat and poultry processing workers (*1*,*2*). We describe COVID-19 among workers in other US food manufacturing and agriculture workplaces and update information on COVID-19 among meat and poultry processing workers.

## The Study

The Centers for Disease Control and Prevention (CDC) collected cumulative aggregate data from state health departments on workers in US food processing, food manufacturing, and agriculture workplaces who had laboratory-confirmed COVID-19 (*5*). Requested data elements included the number and type of workplaces that reported >1 COVID-19 case among workers during March 1–May 31, 2020; the number of workers in affected workplaces; the number, demographics, and symptom status of workers with COVID-19; and the number of COVID-19–related deaths among workers. CDC requested the same information for meat and poultry processing workers and published preliminary data (*1*). Symptom data collection varied by workplace; clinical signs and symptom severity were not requested. None of these data had personal identifying information.

Workplaces were defined by the North American Industry Classification System codes 111 (Crop Production) and 311 (Food Manufacturing) (*6*). Demographic and symptom status proportions were calculated after excluding missing and unknown values. Data on sex were missing for 14.8% of food manufacturing and agriculture workers with COVID-19; on age for 13.4%; on symptom status for 33.6%; and on race and ethnicity for 36.3%. Because characteristics of total worker populations in affected workplaces were not available, we compared the racial and ethnic distribution of workers with COVID-19 to the distribution of all workers in the animal slaughtering and processing industry. CDC determined the investigation to be nonresearch as defined in 45 CFR 46.102(l); Paperwork Reduction Act was waived with respect to voluntary collection of information during a public health emergency (*7*).

Among 50 US states, 36 (72.0%) responded to the CDC inquiry; 33 (91.7%) reported >1 laboratory-confirmed COVID-19 case among food processing, food manufacturing, or agriculture workers during March 1–May 31, 2020. States reported 8,978 cases and 55 (0.6%) deaths among workers in 742 food manufacturing and agriculture workplaces in 30 states (Table 1). Among the 30 states reporting cases, the median number of affected facilities per state was 12 (interquartile range [IQR] 4–30 facilities); among 15 states that reported worker populations in affected workplaces, 8.2% of 30,609 workers received COVID-19 diagnoses. The percentage of workers with COVID-19 ranged from 2.0%–43.5% per state.

**Table 1:** Laboratory-confirmed COVID-19 among workers in food manufacturing and agriculture workplaces in 30 US states, March 1–May 31, 2020*

State†	Type of food manufactured or farmed	No. workplaces affected	No. workers in affected workplaces	Confirmed COVID-19 cases among workers, no. (%)	COVID-19–related deaths, no. (%) ‡
Arkansas	Various	14	NA	68 (–)	1 (1.5)
California§	Fruits, vegetables, dairy, packaged foods, frozen foods, seafood, other	30	NA	518 (–)	2 (0.4)
Colorado	Vegetables, dairy, baked goods, packaged foods, other	19	5,773	443 (7.7)	3 (0.7)
Florida	Vegetables, fruits, spices, other	10	NA	280 (–)	2 (0.7)
Georgia	Blueberry, seasonal fruits, other	6	728	268 (36.8)	0
Idaho	Vegetables	3	559	100 (17.9)	0
Illinois	Fruits, dairy, pizza, packaged foods, other	61	NA	987 (–)	6 (0.6)
Iowa	Eggs, dairy, other	9	1870	391 (20.9)	2 (0.5)
Kansas	Baked goods, fruits, dairy, seasonings, other	13	NA	140 (–)	0
Kentucky	Baked goods, jelly, salad dressing, other	8	NA	53 (–)	1 (1.9)
Louisiana	Seafood, dairy	5	607	264 (43.5)	0
Maine	Seafood	1	65	15 (23.1)	0
Massachusetts	Seafood, baked goods, other	173	NA	859 (–)	4 (0.5)
Minnesota	Fruits, vegetables, baked goods, packaged foods, frozen foods, other	36	9,829	434 (4.4)	4 (0.9)
Missouri	Prepared foods, cereal, corn	4	2,180	144 (6.6)	1 (0.7)
Nebraska	Eggs, milk products, baked goods, frozen foods, other	14	3,348	123 (3.7)	0
New Jersey	Produce	3	515	93 (18.1)	2 (2.2)
North Carolina¶	Fruits, vegetables, packaged foods	16	NA	302 (–)	2 (0.7)
Oregon	Vegetables, fruits, frozen foods, packaged foods, other	22	4,579	211 (4.6)	3 (1.4)
Pennsylvania	Seafood, mushrooms, apples, cheese, eggs, other	91	NA	968 (–)	6 (0.6)
Rhode Island	Seafood, apples, cheese, eggs, other	75	NA	346 (–)	13 (3.8)
South Carolina	Vegetables, fruits, pasta, canned foods, frozen foods, other	11	NA	22 (–)	0
South Dakota	Cheese	1	200	7 (3.5)	0
Tennessee	Vegetables, fruits, other	6	NA	323 (–)	1 (0.3)
Utah	Cherries, dairy, baked goods, candy, other	19	NA	186 (–)	0
Vermont	Cheese	1	300	6 (2.0)	0
Virginia	Eggs	1	50	4 (8.0)	0
Washington	Seafood, mushrooms, vegetables, fruits, pasta, frozen foods	37	NA	755 (–)	1 (0.1)
Wisconsin	Vegetables, dairy, pizza, baked goods, other	52	NA	667 (–)	1 (0.1)
Wyoming	Other	1	6	1 (16.7)	0
Total	Various	742	30,609#	8,978	55

*COVID-19, coronavirus disease; NA, not available; –, percentage not calculated due to missing data. †Arizona, Maryland, Montana, New Hampshire, New Mexico, and North Dakota reported no cases of COVID-19 among workers in food manufacturing and agriculture workplaces. ‡Percentage of deaths among cases. §Data from 7 California counties. ¶Reported cases are among workers and close contacts of workers. #Among 15 of 30 states that reported the number of workers in affected workplaces, 8.2% of 30,609 workers received COVID-19 diagnoses.

**Figure 1:**
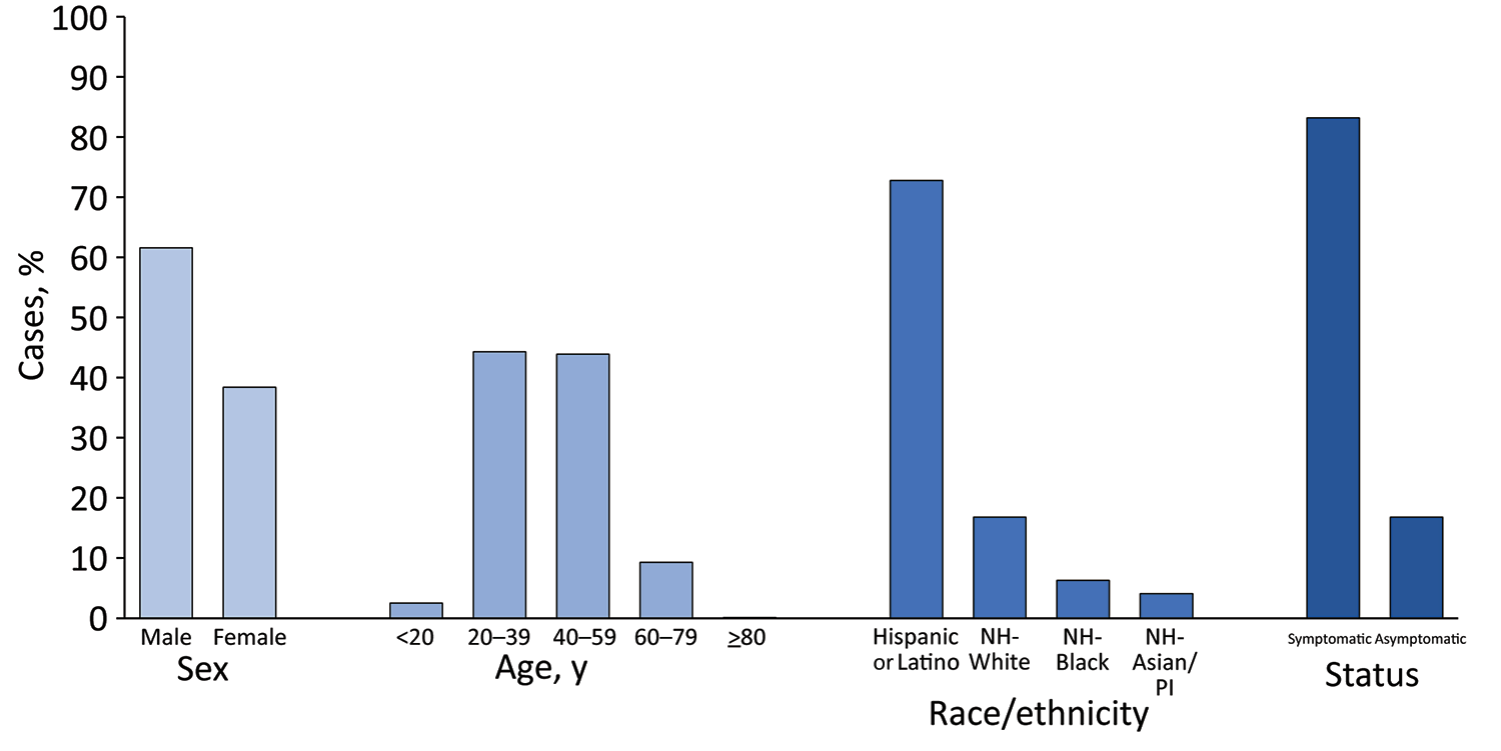
Characteristics of laboratory-confirmed COVID-19 cases among workers in food manufacturing and agriculture workplaces in 28 US states, March 1–May 31, 2020. The analytic dataset includes Arkansas, California, Florida, Georgia, Idaho, Illinois, Iowa, Kansas, Kentucky, Louisiana, Maine, Massachusetts, Minnesota, Missouri, Nebraska, New Jersey, Oregon, Pennsylvania, Rhode Island, South Carolina, South Dakota, Tennessee, Utah, Vermont, Virginia, Washington, Wisconsin, and Wyoming. Characteristics of workers with COVID-19 were not available for 2 states, Colorado and North Carolina. Arizona, Maryland, Montana, New Hampshire, New Mexico, and North Dakota reported no cases of COVID-19 among workers in food manufacturing and agriculture workplaces. The dataset excludes cases among workers for whom information was missing on sex (n = 1,331), age (n = 1,207), race/ethnicity (n = 3,257), and symptom status (n = 3,021). White, Black, and Asian/Pacific Islander workers were non-Hispanic; Hispanic or Latino workers could be of any race. Testing strategies and symptom categorization varied by facility. Symptom status was available for a single timepoint, either the time of testing or the time of interview. Column percentages might not equal 100% due to rounding. COVID-19, coronavirus disease; NH, non-Hispanic; PI, Pacific Islander.

Of cases among food manufacturing and agriculture workers with information on sex (n = 7,647) and age (n = 7,771), 4,713 (61.6%) workers were male, 2,934 (38.4%) were female, and 3,439 (44.3%) workers were 20–39 years of age (Figure 1). Among 5,721 workers with race and ethnicity reported, 4,164 (72.8%) workers were Hispanic or Latino, 963 (16.8%) were non-Hispanic White, 362 (6.3%) were non-Hispanic Black, and 232 (4.1%) were non-Hispanic Asian/Pacific Islander. Overall, 83.2% of cases occurred among racial and ethnic minority workers. Symptom status was reported for 5,957 workers; 4,957 (83.2%) workers were symptomatic and 1,000 (16.8%) were asymptomatic or presymptomatic.

**Figure 2:**
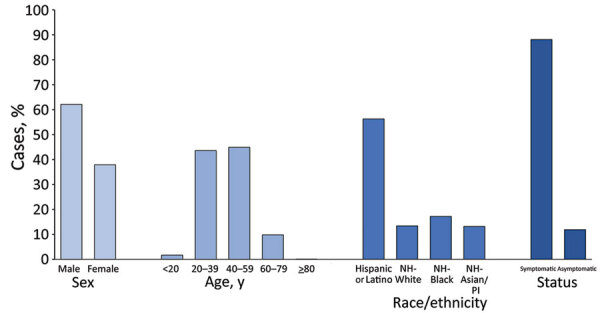
Characteristics of laboratory-confirmed COVID-19 cases among workers in meat and poultry processing facilities in 29 US states, March 1–May 31, 2020. Preliminary data were published in Morbidity and Mortality Weekly Report (*1*); 8 additional states, Arkansas, California, Iowa, Louisiana, Kansas, Minnesota, New Jersey, and Oregon provided data that was not included in the prior assessment. Characteristics of workers with COVID-19 were not available for 2 states, Colorado and North Carolina. Florida, Montana, New Hampshire, North Dakota, and Vermont reported no cases of COVID-19 among workers in meat and poultry processing facilities. The analytic dataset excludes cases among workers for whom information was missing on sex (n = 4,475), age (n = 6,695), race/ethnicity (n = 8,553), and symptom status (n = 8,437). White, Black, and Asian/Pacific Islander workers were non-Hispanic; Hispanic or Latino workers could be of any race. Testing strategies and symptom categorization varied by facility. Symptom status was available for a single timepoint, at the time of testing or at the time of interview. Column percentages might not equal 100% due to rounding. COVID-19, coronavirus disease; NH, non-Hispanic; PI, Pacific Islander.

States reported 28,364 cases and 132 (0.5%) deaths among workers in 382 meat and poultry processing facilities in 31 states (Table 2). Demographic characteristics and symptom status of workers with COVID-19 indicated most were symptomatic and members of racial and ethnic minority groups (Figure 2).

**Table 2:** Laboratory-confirmed COVID-19 among workers in meat and poultry processing facilities in 31 US states, March 1–May 31, 2020*

State†	Type of meat or poultry	No. workplaces affected	No. workers in affected workplaces	Confirmed COVID-19 cases among workers, no. (%)	COVID-19–related deaths, no. (%)‡
Arizona	Beef	1	1,750	162 (9.3)	0
Arkansas	Poultry	49	NA	779 (–)	10 (1.3)
California§	Beef, lamb, pork, poultry, other	11	NA	466 (–)	2 (0.4)
Colorado	Beef, bison, lamb, poultry	7	7,711	422 (5.5)	9 (2.1)
Georgia	Poultry	14	16,500	509 (3.1)	1 (0.2)
Idaho	Beef	2	797	72 (9.0)	0
Illinois	Beef, pork, poultry	26	NA	1,029 (–)	10 (1.0)
Iowa	Beef, pork, poultry	26	22,170	6,131 (27.7)	19 (0.3)
Kansas	Beef, pork, poultry	10	NA	2,670 (–)	8 (0.3)
Kentucky	Pork, poultry	7	7,633	559 (7.3)	4 (0.7)
Louisiana	Poultry	2	1,430	51 (3.6)	0
Maine	Poultry	1	411	50 (12.2)	1 (2.0)
Maryland	Poultry	2	2,036	208 (10.2)	5 (2.4)
Massachusetts	Poultry, other	33	NA	263 (–)	0
Minnesota	Beef, pork, poultry, other	19	15,025	2,120 (14.1)	2 (0.1)
Missouri	Beef, pork, poultry	9	8,469	745 (8.8)	2 (0.3)
Nebraska	Beef, pork, poultry	23	26,134	3,438 (13.2)	14 (0.4)
New Jersey	Beef	1	500	33 (6.6)	0
New Mexico	Beef, pork, poultry	2	550	24 (4.4)	0
North Carolina¶	Pork, poultry	28	32,325	2,491 (7.7)	13 (0.5)
Oregon	Beef, pork, poultry, other	7	1,945	60 (3.1)	0
Pennsylvania	Beef, pork, poultry, other	30	15,548	1,169 (7.5)	8 (0.7)
Rhode Island	Beef, pork, poultry, other	6	NA	78 (–)	0
South Carolina	Beef, pork, poultry, other	16	NA	97 (–)	0
South Dakota	Beef, pork, poultry	4	6,500	1,593 (24.5)	3 (0.2)
Tennessee	Pork, poultry, other	7	NA	640 (–)	2 (0.3)
Utah	Beef, pork, poultry	4	NA	67 (–)	1 (1.5)
Virginia	Pork, poultry, other	14	NA	1,109 (–)	10 (0.9)
Washington	Beef, poultry	7	4,452	468 (10.5)	4 (0.9)
Wisconsin	Beef, pork, veal	14	14,125	860 (6.1)	4 (0.5)
Wyoming#	Beef	0	NA	1 (–)	0
Total	Beef, bison, lamb, pork, poultry, veal, other	382	186,011**	28,364	132

*Preliminary data published in Morbidity and Mortality Weekly Report (*1*); 8 additional states, Arkansas, California, Iowa, Louisiana, Minnesota, New Jersey, North Carolina, and Oregon, provided data that was not included in the prior assessment. COVID-19, coronavirus disease; NA, not available; –, percent not calculated due to missing data. †Florida, Montana, New Hampshire, North Dakota, and Vermont reported no cases of COVID-19 among workers in meat and poultry processing facilities. ‡Percentage of deaths among cases. §Data from 7 California counties. ¶Reported cases are among workers and close contacts of workers. #One worker with COVID-19 worked at a meat processing facility in another state. **Among 20 of 31 states reporting the number of workers in affected workplaces, 11.4% of 186,011 workers received COVID-19 diagnoses.

## Conclusions

We describe COVID-19 among workers in US food processing, food manufacturing, and agriculture workplaces during March 1–May 31, 2020. Among all food manufacturing and agriculture workers in 28 states reporting race and ethnicity data, 36.5% of workers are Hispanic or Latino, 52.6% are non-Hispanic White, 5.9% are non-Hispanic Black, 3.5% are non-Hispanic Asian/Pacific Islander, and 1.5% are of other non-Hispanic race or ethnicity groups (*4*). However, among workers with COVID-19 for whom race or ethnicity data were reported, 72.8% were Hispanic or Latino, 6.3% were non-Hispanic Black, and 4.1% were non-Hispanic Asian/Pacific Islander, suggesting that Hispanic or Latino, non-Hispanic Black, and non-Hispanic Asian/Pacific Islander workers in these workplaces might be disproportionately affected by COVID-19.

The sex, age, and symptom distribution of meat and poultry processing workers with COVID-19 was similar to that observed for food manufacturing and agriculture workers. The racial and ethnic distribution of meat and poultry processing workers with COVID-19 differed slightly; a higher percentage of cases were reported among non-Hispanic Black and non-Hispanic Asian/Pacific Islander workers.

Our study supports findings from prior reports that part of the disproportionate burden of COVID-19 among some racial and ethnic minority groups is likely related to occupational risk (*8*,*9*). These findings should be considered when implementing workplace interventions to ensure communication and training are culturally and linguistically tailored for each workforce.

Reports on mass testing in US meat and poultry processing facilities revealed widespread COVID-19 outbreaks and identified high proportions of asymptomatic or presymptomatic infections (*10*,*11*). Although most food manufacturing and agriculture workers (83.2%) and meat and poultry processing workers (88.1%) in our study reported symptoms, not all workplaces performed mass testing; therefore, workers with asymptomatic or presymptomatic infections might have been missed. These findings support the need for comprehensive testing strategies, coupled with contact tracing and symptom screening, for high-density critical infrastructure workplaces to aid in identifying infections and reducing transmission within the workplace (*12*).

Reducing workplace exposures is critical for protecting workers in US food processing, food manufacturing, and agriculture workplaces and might help reduce health disparities among disproportionately affected populations. Adherence to workplace-specific intervention and prevention efforts, including engineered controls, such as physical distancing; administrative controls, such as proper sanitation, cleaning, and disinfection; and providing personal protective equipment likely would protect both workers and surrounding communities (*13*,*14*).

This study has several limitations. First, only 36 states reported data; these results might not be representative of all US food processing, food manufacturing, and agriculture workers and workplaces. Second, testing strategies varied by workplace, influencing the number of cases detected and reported among workers. Workers might have been hesitant to report illness or seek healthcare, which could have led to underestimating cases among workers. Delays in linking cases and deaths to workplace outbreaks likely also contributed to an underestimation. Third, demographic characteristics of total worker populations in all affected workplaces were not available, limiting the ability to quantify the degree to which some racial and ethnic minority groups might be disproportionately affected by COVID-19. Fourth, preferred language, English proficiency, and migration and immigration status of workers were not captured; culturally and linguistically appropriate public health monitoring and interventions are crucial considerations for this workforce. Finally, workers are members of their local communities; transmission of SARS-CoV-2 could have occurred both at the workplace and in the surrounding community and thus could be affected by levels of community transmission.

Comprehensive evaluations in food processing, food manufacturing, and agriculture workplaces and communities are needed to clarify and address risk factors for SARS-CoV-2 transmission among workers. The extent of control measures and timing of implementations should be evaluated to assess effectiveness of workplace interventions. Several factors at the individual-, household-, community-, and occupational-level, including long-standing health and social disparities, likely contribute to disproportionate disease incidence among racial and ethnic minority workers.
